# Antioxidant Activities of Sorghum Kafirin Alcalase Hydrolysates and Membrane/Gel Filtrated Fractions

**DOI:** 10.3390/antiox8050131

**Published:** 2019-05-15

**Authors:** Shiwei Xu, Yanting Shen, Yonghui Li

**Affiliations:** Department of Grain Science and Industry, Kansas State University, Manhattan, KS 66506, USA; xushiwei@ksu.edu (S.X.); yantings@ksu.edu (Y.S.)

**Keywords:** grain sorghum, kafirin, protein hydrolysate, antioxidant, emulsion, ground meat, peptide sequence

## Abstract

Sorghum has a significant amount of proteins, especially kafirin; however, limited information is available on evaluating its potential for peptide antioxidants. The objectives of this study were to: (1) investigate the effects of two key variables, enzyme-to-substrate ratio and reaction time on kafirin hydrolysis using Alcalase; (2) evaluate the antioxidant performances of the hydrolysates and fractions from membrane ultrafiltration and gel filtration; and (3) identify peptide sequences in the antioxidant fraction using MALDI-TOF/TOF MS. Kafirin hydrolysates prepared at enzyme-to-substrate ratio of 0.4 Au/g and 4 h had a good balance of antioxidant activity, yield, and economic efficiency. Medium-sized fraction of hydrolysates (5–10 kDa) from membrane filtration possessed the highest antioxidant activities among various fractions. The fraction also unveiled a good inhibition effect against lipid oxidation in emulsion and ground meat systems. Smaller-sized fraction (F3) collected through gel-filtration chromatography had significantly stronger antioxidant activities than other fractions, and 26 representative peptide sequences were identified in the fraction.

## 1. Introduction

Sorghum is one of the oldest known ancient grains and is the third largest cereal crop in the United States. It is mainly used as animal feed and a starch source for biofuel [1,2]. There has been an increasing trend of adding values to under-utilized crops, including sorghum. Driven by the heightened safety concerns over synthetic antioxidants and consumers’ preference for natural ingredients, the development of novel antioxidants has drawn growing interests [3,4]. At the same time, protein has been one of the top driving factors in current markets with extensive popularity. Peptide antioxidants are naturally existent (e.g., glutathione, carnosine, anserine), and can also be produced from dietary proteins, which exert antioxidative performances through multiple pathways such as scavenging free radicals, chelating transition metals, reducing oxidized substances, interrupting the decomposition of hydroperoxide, forming physical barriers to hinder the access of pro-oxidant to targets, etc. [5,6]. Antioxidant peptides are naturally-derived, efficient, generally considered as safe at high dosages, and also a valuable source of essential amino acids. Besides, due to the surface amphiphilicity, peptide antioxidants can act as functional ingredients with special properties (e.g., gelation, emulsifying, foaming, water and/or oil binding capacity) [5,7]. Thus, they are promising alternatives to synthetic antioxidants to be incorporated into various food products as additives to protect lipids from oxidation.

Upon enzymatic hydrolysis, native globular matrix of intact proteins is cleaved, characteristic structures (e.g., functional side groups, structural domains) attributing to the antioxidative activities are exposed, and specific peptide sequences are released [8]. The solubility of hydrolyzed proteins is increased, yet, the amino acid profile could remain essentially unchanged or improved in some fractions [9]. The resulting hydrolysates can be purified and isolated by various techniques to obtain peptide fractions with enhanced activities. The selection of enzyme is important in determining the end-use properties of hydrolysates. Alcalase is a commercial protease prepared by submerged fermentation of a selected strain from *Bacillus licheniformis*, with Subtilisin Carlsberg as a major proteolytic component. It is a serine endopeptidase with broad specificity, which cleaves peptide bonds at the interior of the chain and produces peptide with varied sizes [10,11,12]. Alcalase is readily water-soluble at all concentrations, available in food grade, and has a relatively low bulk sale price, which has been widely used in food industry to prepare plant and animal proteins with improved functional and nutritional values [13]. Alcalase, alone or as a partial step, can be used for generation of antioxidative peptides from plant and/or animal proteins [10,11,12,14,15,16,17,18,19,20,21,22]. Xia et al. [10] reported that barley glutelin hydrolysates prepared with Alcalase demonstrated a significantly higher antioxidant capacity including superoxide radical scavenging activity, hydroxyl radical scavenging activity and ferrous iron chelating ability than that prepared with Flavourzyme. Thamnarathip et al. [16] indicated that rice bran protein hydrolysate prepared with Alcalase yielded a higher amount of antioxidative hydrolysates, degree of hydrolysis, and protein content compared to those from Neutrase and Flavourzyme. Pan et al. [21] also found that Alcalase yielded a relatively higher degree of hydrolysis and DPPH quenching activity of antioxidant peptides than Papain, Neutrase, and Protamex [21]. Bioactive peptides prepared with Alcalase were also found to be more resistant to digestive enzymes [14,15].

Sorghum and its byproduct (e.g., distillers’ grains) have a significant amount of proteins, especially kafirin; however, very limited studies have yet been available on evaluating its potential to be employed as peptide antioxidants [17,23,24,25,26,27]. Therefore, the objectives of this study were to: (1) investigate the effects of reaction variables on kafirin hydrolysis using Alcalase; (2) evaluate the antioxidant performances of kafirin Alcalase hydrolysates and fractions from membrane ultrafiltration as well as gel filtration by chemical assays and food models; and (3) identify peptides sequences in the antioxidant peptide fraction using reverse phase high performance liquid chromatography (RP-HPLC) and matrix-assisted laser desorption ionization-time of flight/time of flight mass spectrometry (MALDI-TOF/TOF MS).

## 2. Materials and Methods

### 2.1. Materials

White sorghum flour (9.33% protein) was obtained from ADM Milling Co. (Overland Park, KS, USA). Alcalase^®^ 2.4L (Proteinase from *Bacillus licheniformis Subtilisin A*) was purchased from Sigma-Aldrich (St. Louis, MO, USA). 2,2-Diphenyl-1-picrylhydrazyl (DPPH), 2,2′-Azino-bis (3-ethylbenzothiazoline-6-sulfonic acid) diammonium salt (ABTS), 2,2′-Azobis (2-methylpropionamide) dihydrochloride (AAPH), fluorescein, 6-hydroxy-2,5,7,8-tetramethylchroman-2-carboxylicacid (Trolox), Folin & Ciocalteu phenol reagent, sodium tetraborate decahydrate (Na_2_B_4_O_7_), and cumene hydroperoxide were from Sigma-Aldrich (St. Louis, MO, USA). Potassium ferricyanide (K_3_[Fe(CN)_6_]) and l-serine were from Acros Organics (New Jersey, USA). Sodium dodecyl sulfate (SDS), trichloroacetic acid (TCA), o-phthaldialdehyde (OPA), and dithiothreitol (DTT) were acquired from Thermo Fisher Scientific Inc. (Ottawa, ON, USA).

### 2.2. Preparation of Sorghum Protein Hydrolysates

Kafirin protein was isolated from defatted white sorghum flour by glacial acetic extraction method [28] and was freeze-dried (Freezone 4.5, Labconco Corporation, Corneous City, MO, USA) and ground for later use. The protein content of extracted kafirin was determined to be 97.31% by nitrogen combustion analysis using LECO FP-2000 nitrogen analyzer (St. Joseph, MI, USA) with nitrogen conversion factor of 6.25. In conical flasks, protein suspensions (4%, w/w) were prepared by dispersing 1 g kafirin in 25 mL deionized water. The suspensions were then adjusted to pH 8.3 with 1 N NaOH, and added with 0.083, 0.167, or 0.333 g of Alcalase (2.4 Au/g), respectively, to achieve final enzyme-to-substrate ratios of 0.2, 0.4, and 0.8 Au/g. The flasks were sealed with rubber stoppers and transferred to a 50 °C water bath shaker (Shel Lab SWBR27, VWR International, LLC., Radnor PA, USA). At all enzyme levels, hydrolysis with varied reaction times was conducted by incubating for 0.5, 1, 2, 3, 4, 5, 6, 8, and 11 h followed with heating in a boiling water bath for 15-20 minutes, respectively. After cooling down, the solutions were adjusted to pH 7.0 with either 1 N HCl or 1 N NaOH and centrifuged at 3500× *g*, 4 °C for 25 min. The supernatant (i.e., hydrolysate) was collected, lyophilized, and stored at −4 °C till further analysis.

### 2.3. Hydrolysate Fractionation and Peptide Identification

#### 2.3.1. Ultrafiltration with Stirred Cell

The ultrafiltration was achieved using an Amicon^®^ Stirred Cell (EMD Millipore Corporation, Billerica, MA, USA) sequentially assembled with Ultracel^®^ Ultrafiltration Discs (EMD Millipore Corporation, Billerica, MA, USA) with molecular weight cut-offs (MWCO) at 10 k, 5 k, 3 k and 1 kDa. New membranes were floated in deionized water with skin (glossy) side adown for 24 h with water changed at least three times to remove the pretreated glycerine residues. The membrane was then placed into the stir cell and loaded with hydrolysate solution at 5 mg/mL. The stir cell was placed on top of a magnetic stirrer at 60 rpm. Compressed nitrogen was connected to the stir cell at a maximal pressure of 60 psi to accelerate the penetration process.

#### 2.3.2. Gel Filtration Chromatography

The hydrolysate (100 mg) collected from ultrafiltration was dissolved in 3 mL deionized water and loaded onto a Sephadex G-25 (medium) gel filtration column (26 mm × 850 mm) which had been previously equilibrated with deionized water. A total portion of 600 mL deionized water was used to elute the sample, and aliquots of 3 mL were manually collected at a gravity-driven flow. The aliquots were measured for their absorbance at 280 nm using a UV/Vis spectrophotometer (UV-6300PC, VWR International, LLC., Radnor PA, USA) to determine the elution profile, and were combined into three major fractions (F1–F3) based on the chromatogram. The three gel filtrated fractions were lyophilized and analyzed, respectively.

#### 2.3.3. Identification of Kafirin Proteins and Peptide Sequences

Crude extracted kafirin was dissolved in 100 μL DTT (15 μg/mL) for 30 min at 80 °C. 100 μL iodoacetamide (18 μg/mL) was then added. The mixture was placed in the dark and incubated for 1 h at room temperature. The proteins were then digested with 10 µL of trypsin (1 µg/30 µL, Trypsin Gold, mass spectrometry grade; Promega Corp., Madison, WI) overnight at 37 °C. Digested kafirin solutions were spotted in a 2,5-dihydroxybenzoic acid (DHB) matrix (Sigma-Aldrich, St. Louis, MO, USA) on a Bruker Ultraflex III MALDI-TOF/TOF MS (Bruker Daltonik GmbH, Bremen, Germany). Spectra were obtained in positive ion reflection mode at 66.7 Hz with 1000 laser shots per spectrum. Spectra were analyzed using FlexAnalysis (version 3.3, Bruker Daltonik GmbH) and internally calibrated with DHB matrix peaks. Known alpha, beta, and gamma kafirin protein sequences were obtained from http://www.uniprot.org. Using mMass software, these sequences were analyzed allowing for up to 4 missed cleavages, a peptide mass tolerance of 0.55 Da, and variable modifications of carbamidomethyl (C) and oxidation (W, M). The spectrum from the trypsin cleavage of the proteins was compared to the known theoretical sequence cleavage results.

Samples of lyophilized fractions from gel filtration were analyzed using RP-HPLC on a Beckman machine running 32 Karat (version 8.0) software with a C8 column (buffer A: 99.9% water and 0.1% TFA; solvent B: 90% acetonitrile, 9.9% water, and 0.1% TFA) with a 10 to 50% buffer B gradient over 30 min. Peaks from the RP-HPLC runs were manually collected and analyzed using the same MALDI-TOF/TOF MS procedure as described for crude kafirin. The spectra obtained were compared to beta kafirin protein sequences (http://www.uniprot.org). These protein sequences were cleaved using non-specific cleavage sites that resulted in peptides between 500 and 3000 Da, and masses were compared to the spectra from the HPLC peaks using a peptide mass tolerance of 0.55 Da.

### 2.4. Characterization of Protein Hydrolysate and Antioxidant Properties

#### 2.4.1. Total Protein Recovery

Percentage of water-soluble hydrolysate yield was calculated as total protein recovery and used to determine the efficiency of the hydrolysis process [9]. Total protein recovery (i.e., hydrolysate yield) was determined by the percentage of the hydrolysates to the initial protein by excluding the unhydrolyzed protein obtained through centrifuging the resulting reaction mixture:

$$\mathrm{Total}\;\mathrm{protein}\;\mathrm{recovery}\%=\frac{W_{i}-W_{p}}{W_{i}}\times 100\%\;$$

where W_i_ was the weight of initial protein (g), and W_p_ was the weight of lyophilized precipitate (g) obtained from centrifuging the resulting mixture at the end of hydrolysis.

#### 2.4.2. Degree of Hydrolysis

Degree of hydrolysis (DH) is defined as the percentage of hydrolyzed peptide bonds to the number of total bonds per unit weight of the substrate protein, which is a typical indicator of the extent of hydrolysis degradation. DH was determined using the previously used OPA method [29]. 

#### 2.4.3. Total Phenolic Content

The total phenolic content (TPC) was determined following the Folin–Ciocalteu procedure [16]. Briefly, 1 mL 1:10 (v/v) Folin–Ciocalteu reagent and 3 mL 7.0% (w/w) Na_2_CO_3_ was sequentially added to 1 mL of sample solution at 1.0 mg/mL. The absorbance of the reaction mixture was measured at 760 nm after being incubated for 30 min in darkness at room temperature. The total phenolic content in sample was expressed as mg gallic acid equivalents per gram of sample (mg GAE/g).

#### 2.4.4. DPPH Radical Scavenging Activity

DPPH radical scavenging activity (DPPH%) was determined by the percentage of decrease in DPPH radical concentration. Briefly, 0.02 mM DPPH reagent was prepared freshly by dissolving 7.88 mg of DPPH in 100 mL 95% (v/v) ethanol. 4.8 mL of sample at varied concentration (1–10 mg/mL) was mixed with equal volume of DPPH reagent. The reaction mixture was incubated in darkness for 30 min at room temperature before reading absorbance at 517 nm. The reduction of DPPH radicals by samples was expressed as percentage of decrease in absorbance at 517 nm as compared to a blank control [22]. DPPH radical scavenging activity was calculated as:

$$\mathrm{DPPH}\%=\frac{A_{b}\;-{\;A}_{s}}{A_{b}}\times 100\%$$

where A_b_ was the absorbance of blank and A_s_ was the absorbance of sample.

#### 2.4.5. ABTS Radical Scavenging Activity

The ABTS radical scavenging activity (ABTS%) of protein hydrolysates or peptides was determined according to the method of Alashi et al. with modifications [9]. The ABTS stock solution was made by mixing equal amount of 7.4 mM ABTS solution and 2.6 mM potassium persulfate solution and stored for 12–16 h in dark at room temperature to generate ABTS radicals. The ABTS stock solution was then diluted with deionized water to achieve an absorbance of 1.1 ± 0.02 at 734 nm on a spectrophotometer. 2.85 mL of the diluted ABTS radical solution was mixed with 0.15 mL of sample solution and incubated at room temperature in darkness for 10 min before reading its absorbance at 734 nm. Deionized water was used as a blank control. ABTS% was calculated using follow equation:

$$\mathrm{ABTS}\%=\frac{A_{b}\;-{\;A}_{s}}{A_{b}}\times 100\%$$

where A_b_ was the absorbance of blank and A_s_ was the absorbance of sample.

#### 2.4.6. Oxygen Radical Absorbance Capacity

Oxygen radical absorbance capacity (ORAC) assay measures the effect of antioxidant on delaying the decline of fluorescence induced by a peroxyl radical generator, AAPH. The assay was performed according to a previous protocol [30] using a Biotek^®^ Synergy H1 Hybrid Microplate Reader (Winooski, VT, USA). Fluorescein was used as the fluorescent probe. The fluorescence of the reaction mixture was recorded every minute for 2 h at 37 °C with excitation and emission wavelengths of 485 and 528 nm, respectively. ORAC values for samples were expressed as gram of Trolox equivalent per gram of sample (g Trolox equiv./g) by comparing the relative area under the sample curve to the Trolox standard curve.

#### 2.4.7. Ferric Ion Reducing Power

The reducing power assay measures the ability of the antioxidant to reduce ferric ion to ferrous ion, which indicates the antioxidant’s capacity in donating an electron or hydrogen [10,31]. Briefly, hydrolysates dissolved in 4 mL 0.2 M phosphate buffer (pH = 6.6) were added with 4 mL 1% (w/v) potassium ferricyanide (K_3_[Fe(CN)_6_]). The mixture was incubated at 50 °C for 20 min, after which 4 mL of 10% (w/v) trichloroacetic acid was added. The reaction mixture was centrifuged at 3500× *g*, 20 °C for 15 min and 4 mL of the supernatant was transferred. Finally, 4 mL of deionized water and 0.8 mL of 0.1% (w/v) ferric chloride were added to the reactant supernatant. After 10 min incubation at room temperature, the absorbance of the resultant mixture was measured at 700 nm. A larger increased absorbance of the sample over blank indicates a stronger reducing power [10,22].

#### 2.4.8. Metal Chelating Capacity

The metal chelating capacity was determined according to the method described by Decker and Welch (1990) with some modifications [32]. Briefly, 1 mL of sample solution at different concentrations (1–10 mg/mL) was pre-mixed with 0.05 mL of 2 mM FeCl_2_ solution. Then, 2 mL of deionized water was added to the mixture and the solution was mixed vigorously on a Vortex mixer (Vortex-Genie 2, Scientific Industries, Inc., Bohemia NY, USA). 0.1 mL ferrozine solution at 5 mM was added to the previous reaction mixture. The absorbance of the final solution was measured at 562 nm after 10 min incubation at room temperature. The metal chelating ability was calculated as:

$$\mathrm{Metal}\;\mathrm{Chelating}\%=\frac{A_{c}-{\;A}_{s}}{A_{c}}\times 100\%$$

where A_s_ and A_c_ represent the absorbance of sample and control, respectively [8]. 

#### 2.4.9. Inhibition of Lipid Oxidation in Oil-in-Water Emulsion Systems

Oil-in-water emulsion samples were prepared according to a previously described method with modifications [7]. Briefly, 250 and 500 mg of selected fraction of kafirin hydrolysates were dispersed in 45 mL of 0.1 M phosphate buffer (pH = 7.0) in 100 mL screw-capped bottles, respectively. The protein solutions were sequentially added with 5 mL soy oil and 0.45 mL Tween 20. The final concentrations of hydrolysates were 50 and 100 mg per mL of soy oil (50 & 100 mg/mL), respectively. The mixture was blended with a homogenizer (PowerGen 700, Fisher Scientific Inc., Ottawa ON, USA) for 2 min followed by passing through a high-pressure microfluidizer (Microfluidics Corp, MA, USA) twice at 30,000 psi to obtain fine emulsions. For comparison, a control containing all the reagents except for kafirin hydrolysates was also prepared under the same emulsifying conditions. The emulsion turbidity and stability were determined [7]. 25 µL of the obtained fine emulsion was transferred to 7 mL of 0.1% SDS solution immediately after the emulsion was formed, and the absorbance of the mixture was monitored at 500 nm on a spectrophotometer. After 180 min, this value was monitored again using the same procedure. The absorbance at time 0 min was interpreted as emulsion turbidity. The emulsion stability was defined as the percentage of turbidity ratio of 180 min to 0 min.

The three obtained emulsions (blank, 50 mg/mL, and 100 mg/mL) were transferred to 50 mL screw-capped tubes and were incubated in a dark in an isothermal oven at 37 °C for autoxidation. A few drops of 3 mM sodium azide were added as a microbial preservative, and the oxidative stabilities were evaluated by measuring the accumulation of hydroperoxides (POV) and thiobarbituric acid reactive substances (TBARS) at 0, 2, 4, 6, 8, 10, 12, and 14 days of incubation, respectively. The POV was determined using a ferric thiocyanate method [7,33]. TBARS was determined by mixing 0.3 mL of the incubated emulsion sample with 0.7 mL of deionized water and 2 mL of TBA reagent containing 15 g of trichloroacetic acid (TCA), 0.375 g of 2-thiobarbituric acid (TBA), and 1.76 mL of 12 N HCl in 82.9 mL of deionized water. The mixture was heated in a boiling water bath for 15 min and then cooled to room temperature for 10 min in a cool water bath. The mixture was then centrifuged at 3500× *g* for 15 min and the absorbance of upper layer was determined at 532 nm after 10 min standing at room temperature. The concentration of TBARS was calculated against a standard curve prepared with 1,1,3,3-tetramethoxypropane and expressed as µM tetramethoxypropan equivalent.

#### 2.4.10. Inhibition of Lipid Oxidation in Meat Systems

Selected fraction of kafirin hydrolysates with promising antioxidant activities was further evaluated for their performance against lipid peroxidation in a ground pork model system according to a described procedure [18]. Ground pork containing two different concentrations of antioxidant peptides were prepared, i.e., 0.5 and 1.0 mg hydrolysate per gram of meat, respectively. Sample containing no antioxidant was also prepared similarly for comparison. The prepared samples were stored at 4 °C refrigerator for different periods (i.e., 0, 1, 2, 4, 6, 8, 10, and 12 days), and the extent of lipid peroxidation was quantified by measuring the TBARS [18]. 

### 2.5. Statistical Analysis

The data were analyzed using SAS software version 9.3 (SAS Institute, Cary NC, USA). Results were evaluated by one-way analysis of variance (ANOVA). Tukey’s post-hoc test was used to assess the significant differences among individual data sets. The results were illustrated as means ± standard deviation (*n* = 3) and were considered as significant at *p* < 0.05.

## 3. Results and Discussion

### 3.1. Effects of Reaction Time and Enzyme-to-Substrate Ratio on Hydrolysis Process and Antioxidant Activity

Kafirin was hydrolyzed with Alcalase at 0.2, 0.4, and 0.8 Au/g enzyme-to-substrate ratios with varied hydrolysis times from 0.5 to 11 h. Total protein recovery, degree of hydrolysis, total phenolic content, and DPPH radical scavenging activities were evaluated for the resulting hydrolysates with these combined treatments. 

**Figure 1:**
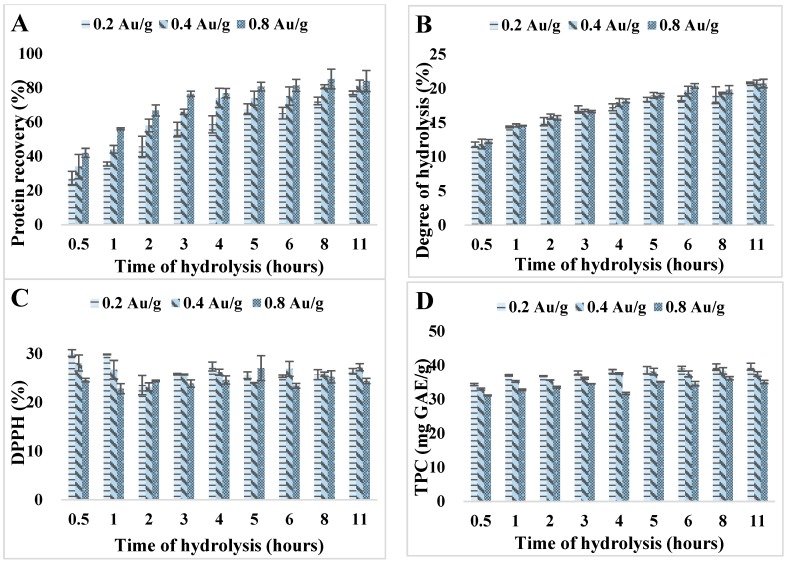
Reaction optimization of antioxidant kafirin Alcalase hydrolysates prepared at combined hydrolysis time of 0.5 to 11 h and enzyme-to-substrate ratios of 0.2, 0.4, and 0.8 Au/g. (**A**). Total protein recovery (%); (**B**). degree of hydrolysis; (**C**). DPPH scavenging activity (%) at 5 mg/mL; (**D**). total phenolic content (mg GAE/g).

The total protein recovery increased dramatically with extended hydrolysis time (Figure 1A). After 2 h of hydrolysis, the yields of hydrolysates reached almost 50% for all enzyme treatments. After 4 h, 0.4 Au/g enzyme treatment exceeded 74% recovery, which indicated that most high *M*_w_ proteins were degraded into low *M*_w_ soluble peptides through peptide bond cleavage. At 4 h of reaction time, an obvious improvement in total protein recovery was observed when increasing the enzyme level from 0.2 to 0.4 Au/g. 

Besides, degree of hydrolysis increased consistently with prolonged hydrolysis time until reaching a plateau at around 4 h (Figure 1B). It was believed that at initial stage of protein hydrolysis, the substrate concentration as well as the enzyme activity were relatively higher, leading to a higher rate of peptide bond cleavage and proteolysis [34]. As the hydrolyzing reaction progressed, the rate decreased when the system tended to reach a reaction equilibrium.

DPPH is a stable free radical with an intrinsic purple color that can be detected at 517 nm. When encountering an electron-donating substrate, it accepts an electron and becomes a stable diamagnetic molecule hence loses the purple color [22]. Therefore, the ability of antioxidants to act as electron donors can be evaluated by DPPH radical scavenging activity. DPPH% of kafirin hydrolysates became stable at around 23.89–27.23% at 5 mg/mL after 3 h of hydrolysis (Figure 1C). The abnormal high value of DPPH% at the beginning state of hydrolysis (0.5 h) might be due to the intrinsic antioxidant activity of the protease. All other hydrolysates exhibited similar values of DPPH% and no obvious increase of DPPH% was observed when longer hydrolysis time was applied. It was interpreted that extended hydrolysis time increased the total protein recoveries but not necessarily the antioxidant activities.

Total phenolic content quantifies total amount of phenolic compounds including phenolic peptides within the hydrolysates, which is an important indicator of hydrolysate compositions. TPC measured for kafirin hydrolysates was between 31.13 and 39.47 mg GAE/g (Figure 1D). While TPC increased with hydrolysis time extended from 0.5 to 2 h for all three enzyme-to-substrate ratios, increasing reaction time from 4 to 11 h did not make a huge impact on TPC. Thamnarathip et al. also reported that increasing hydrolysis time from 2 to 6 h did not significantly change TPC of rice bran protein hydrolyzed by Alcalase [16]. Meanwhile, Liu et al. determined that TPC of defatted wheat germ hydrolysates continuously elevated throughout the fermentation hydrolysis within 24 h, due to the changed pattern of polyphenol-protein interaction and release of free soluble polyphenols, which consequently enhanced the antioxidant activities [35].

Above all, reaction time plays a crucial role in determining the peptide lengths, M_w_, amino acid compositions, and ultimately antioxidant activities of the protein hydrolysates. In the study of Thamnarathip et al., it was observed that large-sized peptides over 40 kDa were completely degraded after 2 h of hydrolysis as unveiled by SDS-PAGE patterns, and the large bands shown in 2-h hydrolysates disappeared after 4 h of hydrolysis. In another study of Kong and Xiong (2006), the three main components in zein protein were completely digested and only trace amount of proteins were left after 5 h hydrolysis with Alcalase [11]. They further indicated that if the hydrolysis became too extensive (>4 h), it could reduce the peptides’ ability to act as physical barrier to prevent oxidants from reaching the lipid fraction in the liposome [11]. 

Overall, kafirin hydrolysates prepared with Alcalase at enzyme-to-substrate ratio of 0.4 Au/g and hydrolysis time of 4 h had a good balance in protein recovery, degree of hydrolysis, antioxidative activity, and production cost. Therefore, hydrolysates prepared at this combined treatment was selected for further in-depth characterizations and analyses.

### 3.2. Ultrafiltration of Kafirin Alcalase Hydrolysates

**Figure 2:**
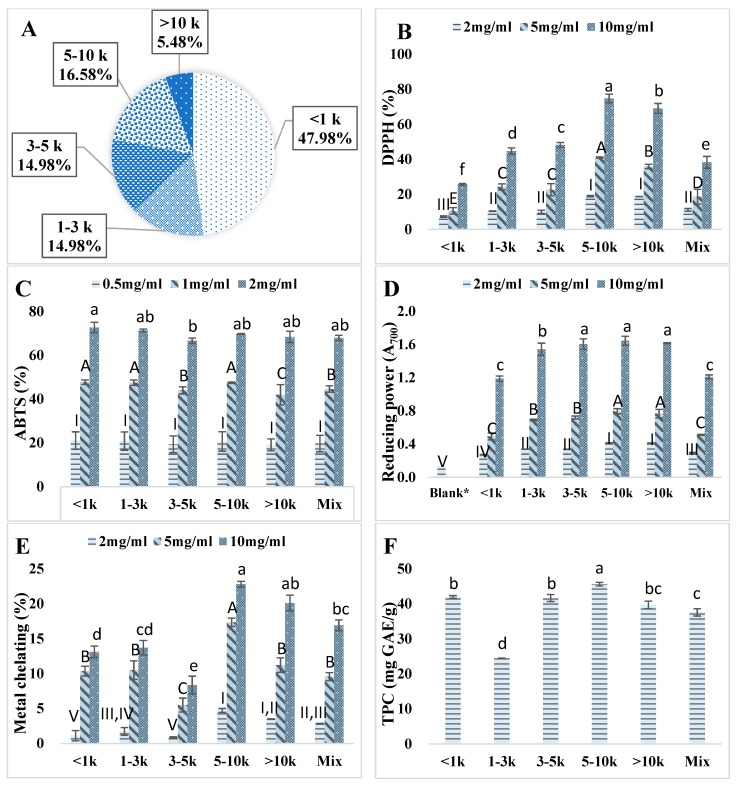
Ultrafiltration of kafirin Alcalase hydrolysates prepared at enzyme-to-substrate ratio of 0.4 Au/g and hydrolyzed for 4 h followed with MWCO membrane filtration at 10 k, 5 k, 3 k, and 1 kDa. (**A**). Weight distribution; (**B**). DPPH scavenging activity (%); (**C**). ABTS scavenging activity (%); (**D**). Reducing power capacity (Abs at 700 nm); (**E**). Metal chelating capacity (%); (**F**). Total phenolic content (mg GAE/g). * Blank represents the absorbance of reaction mixture at 700 nm using distilled water in substitute of sample. Different lowercase letters, capital letters, and roman numerals indicated significant difference at *p* < 0.05.

Previous studies revealed that the antioxidative capacity of hydrolysate peptides is dependent on their *M*_w_ distribution [10,36]. Kafirin Alcalase hydrolysate prepared at enzyme-to-substrate ratio of 0.4 Au/g and reaction time of 4 h was sequentially fractionated with MWCO membranes of 10 k, 5 k, 3 k, and 1 kDa. Figure 2A showed the *M*_w_ distribution profile of the five fractions. The yield of hydrolysate in <1 kDa fraction (47.98%) was much higher than others. The 1–3 kDa (14.97%), 3–5 kDa (14.98%), and 5–10 kDa (16.58%) fractions shared similar portions, while the > 10 kDa fraction had the lowest percentage (5.48%). Xia et al. also found that hydrolysates obtained by hydrolysis with Alcalase contain mostly small- and medium-sized peptides, due to the interior cleavage property of this endo-protease [10]. Similar results were reported by Alashi et al. that <1 kDa took up the largest portion followed with 1–3 k, 3–5 k, and 5–10 kDa of hydrolysates obtained from canola protein hydrolyzed with Alcalase [9]. 

Antioxidant potentials of all the five *M*_w_ fractions were measured. The 5–10 kDa (74.74% at 10 mg/mL) fraction had significantly (*p* < 0.05) higher DPPH% while the <1 kDa fraction (25.70% at 10 mg/mL) had the lowest. The scavenging activity was found to be linearly dose-dependent. These results revealed that the free radical scavenging activity is closely related to the *M*_w_ distribution of the substrate hydrolysates [37]. Medium sized kafirin hydrolysates (5–10 kDa) were found to retain higher DPPH scavenging activity which might be because the substrates in 5–10 kDa contain more efficient electron donors and were thus able to stabilize free radicals to less reactive products [38]. 

ABTS radical scavenging activity is another widely used assay to test the hydrogen donating ability of compounds in evaluation of the antioxidative activity of a substrate [37]. As shown in Figure 2C, all of the hydrolysate fractions and mixtures displayed excellent ABTS% (over 66% at 2 mg/mL). The ABST scavenging activity is also a dose-dependent assay that ABTS% increased accordingly with an increased substrate concentration. 

Ferric ion reducing power assay is a typical electron-transfer method involving one redox reaction, where the compound reduces Fe^3+^ to Fe^2+^ [16]. The ability of the peptides in this case is associated with the exposure of electron-dense amino acid side chain groups [16]. From Figure 2D, all fractions of kafirin hydrolysates resulted in significant (*p* < 0.05) higher absorbance values (A_700_ > 1.18 at 10 mg/mL) over blank control (A_700_ = 0.136 ± 0.048), which indicated an excellence in reducing power of kafirin hydrolysate. Overall, medium- (5–10 kDa) and large-sized (>10 kDa) hydrolysates were found to be stronger reducers. Xia et al. also found that the larger-sized fraction (>10 kDa) of barley glutelin hydrolysates had higher reducing power than the smaller-sized fraction (<1 kDa) [10].

No food system can be considered free of metal ions [31]. Transition metals such as Fe^2+^ and Cu^2+^ are well-known catalysts of lipid peroxidation chain reactions [35,37]. Thus, the chelation of metal ion is an important pathway of antioxidant action. In this assay, after adding antioxidant to ferrous chloride, the un-chelated ferrous irons were determined through measuring the formation of ferrous iron ferrozine complex at 562 nm. After reaction, a lower absorbance indicated a higher metal chelating ability. As shown Figure 2E, kafirin Alcalase hydrolysates exhibited metal chelating ability ranging from 8.36 to 22.78% at 10 mg/mL. It appeared that the 5–10 kDa fraction had significantly (*p* < 0.05) higher chelating activity than other fractions especially at lower dosages. This result indicated that kafirin Alcalase hydrolysates demonstrated iron binding capacity, which may be related to its action as peroxidation protector. The ferrous ion chelating ability may also contribute to their hydroxyl radical scavenging effects of antioxidants due to the combined effects [10]. 

All the ultrafiltrated fractions as well as the hydrolysate mixture were evaluated for total phenolic content as shown in Figure 2F. Except for the 1–3 kDa fraction with a very low TPC (24.42 ± 0.10 mg GAE/g), all other hydrolysate fractions exhibited higher TPC values than the hydrolysate mixture (37.57 ± 0.10 mg GAE/g). The uneven distribution of TPC indicates the successful separation of kafirin hydrolysates based on molecular weight profile. Liu et al. claimed that TPC had a strong positive correlation (R^2^ = 0.972) with antioxidant activity measured by DPPH% in wheat germ hydrolysates [35]. However, this relationship was not observed in our study, which may indicate that the antioxidant activities found in kafirin hydrolysates were originated from both phenolic peptides and compounds and other types of peptides.

Overall, the medium-sized kafirin Alcalase hydrolysates especially the 5–10 kDa fraction displayed promising antioxidant activities based on various assays with a good recovery.

### 3.3. Inhibition of Lipid Oxidation in Model Systems

#### 3.3.1. Oil-in-Water Emulsion System

**Figure 3:**
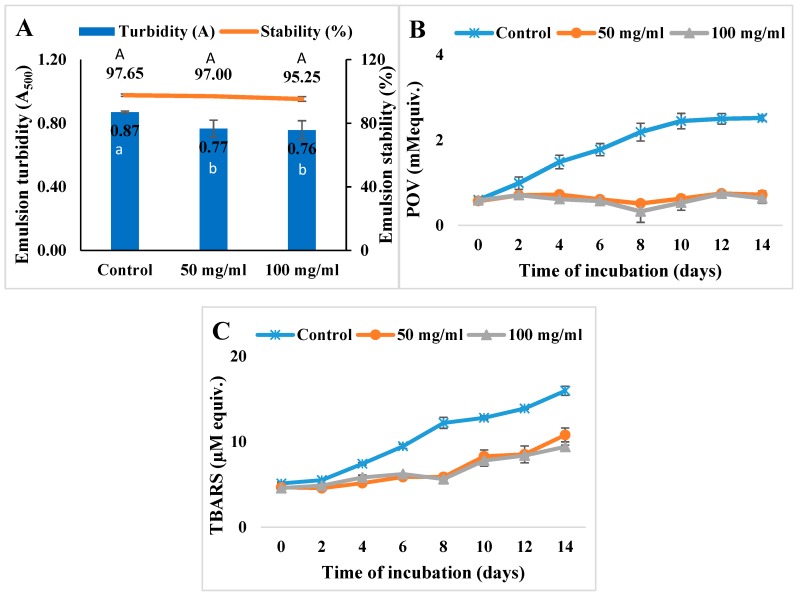
Inhibition effects of kafirin Alcalase 5–10 kDa hydrolysates prepared at enzyme-to-substrate ratio of 0.4 Au/g and hydrolyzed for 4 h in an oil-in-water emulsion model system incorporated at 50 and 100 mg/mL oil. (**A**). Emulsion turbidity (Abs at 500 nm) and stability (%); (**B**). POV (mM cumene hydroperoxide equivalent); (**C**). TBARS (µM tetramethoxypropan equivalent). Different lowercase and capital letters indicated significant difference at *p* < 0.05.

Oil-in-water emulsions exist in many food products such as soups, sauces, beverages, etc. [39]. Oxidation of emulsions is a common problem that can cause texture alteration, development of rancid odor, and loss of nutrition profiles. The kafirin Alcalase 5–10 kDa hydrolysate was evaluated for its ability to improve the oxidative stability of emulsions. Changes in emulsion texture and structure were tested by measuring the emulsion turbidity and stability. As shown in Figure 3A, the addition of hydrolysates at 50 mg and 100 mg hydrolysates per mL of soy oil decreased the emulsion turbidity to A_500_ = 0.77 ± 0.053 and A_500_ = 0.76 ± 0.059, respectively, from A_500_ = 0.87 ± 0.007. Meanwhile, the emulsion stability was not significantly different among the samples. 

The presence of kafirin hydrolysates displayed inhibition effects regarding the formation of both primary and secondary oxidation products, as illustrated in Figure 3B,C. During 14 days of incubation period at 37 °C, the emulsion sample with addition of 50 mg/mL hydrolysates displayed an inhibition rate of POV up to 76.56% (day 8) and had an average inhibition of 55.38%. Where the inhibition rate of TBARS was at the highest of 51.89% (day 8) and was averaged to be 31.70%. Increased concentration of hydrolysates at 100 mg/mL displayed an increase of inhibition effect on oxidation, where the POV inhibition rate was up to 85.02% (day 8) and had an average inhibition value of 58.13%, and TBARS was decreased by 54.03% in maximum (day 8) and averaged at 31.55%. These results were comparable to the previously reported values of potato protein hydrolysates, which were 62.8% and 52.3% inhibition for POV and TBARS, respectively, during the 10 day incubation at 20 mg/mL [39]. 

These results demonstrated the antioxidative activities of fractionated kafirin hydrolysates in decreasing lipid hydroperoxide and TBARS formation, which in turn stabilized the emulsions and retarded the emulsion oxidations. The activities of the hydrolysates could be due to multiple mechanisms. Their free radical scavenging activities, chelation of transition metal ions, reducing power, and interrupting the decomposition of hydroperoxide into secondary products thus inhibiting TBARS formation all contributed to the overall antioxidant capacity [7,11]. Besides, proteins and peptides at oil-water interfaces could form a physical barrier to protect the interior of oil droplets and hinder the access of prooxidants in the aqueous phase.

#### 3.3.2. Ground Meat System

**Figure 4:**
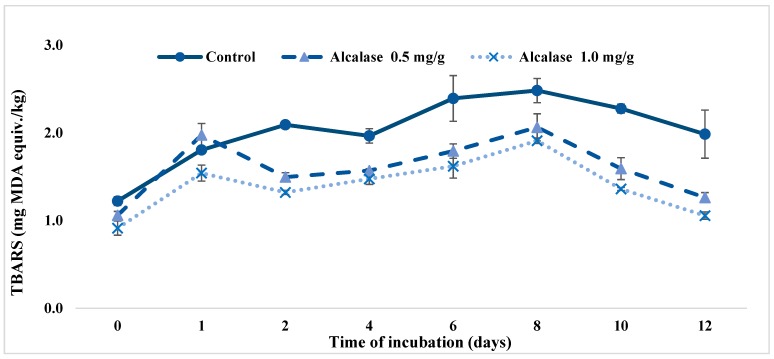
Inhibition effects of kafirin Alcalase 5–10 kDa hydrolysates, measured as TBARS (mg malonaldehyde equivalent per kg of sample), prepared at enzyme-to-substrate ratio of 0.4 Au/g and hydrolyzed for 4 h in a ground meat model system incorporated at 0.5 and 1 mg/g meat.

Reducing hydrogen and lipid peroxides in food products are important reactions because they are able to form carbonyl molecules associated with rancid aroma and form free radicals [5]. The lipid oxidation process in food products is highly dependent on the presence of pro-oxidants such as lipoxygenase, singlet oxygen molecules and transition metals [7]. Concentration of secondary oxidation product TBARS was measured for meat samples incorporated with kafirin 5–10 kDa Alcalase hydrolysates and was compared with a blank control (Figure 4). With antioxidant hydrolysates added at 0.5 and 1.0 mg per gram of meat, TBARS was reduced throughout the entire 12-day incubation period. At day 0, all meat samples had similar TBARS content, and from day 2, the antioxidant hydrolysates started to exhibit apparent oxidation inhibition effects. At the end of the incubation, the TBARS was decreased by 35.34% and 43.17% for 0.5 and 1.0 mg/g samples, respectively, which indicated that the oxidation activities of lipid in ground meat were markedly inhibited by the addition of the kafirin hydrolysates. The average inhibition of TBARS during the 12-day incubation was 26.18 ± 7.06% and 46.92 ± 15.98% for 0.5 and 1.0 mg/g meat samples, respectively. Overall, the inhibition rates of TBARS in this study were higher than that of alkaline protease treated soy protein hydrolysates, which was 21.8% at day 8 and 20.1% at day 15 of storage, when treated at 0.8 mg/g [18]. This experimental data served as important evidence illustrating the antioxidant effects of fractionated kafirin Alcalase hydrolysates in real food products. 

### 3.4. Purification and Identification of Antioxidative Peptides from Kafirin Hydrolysates

#### 3.4.1. Gel Filtration

**Figure 5:**
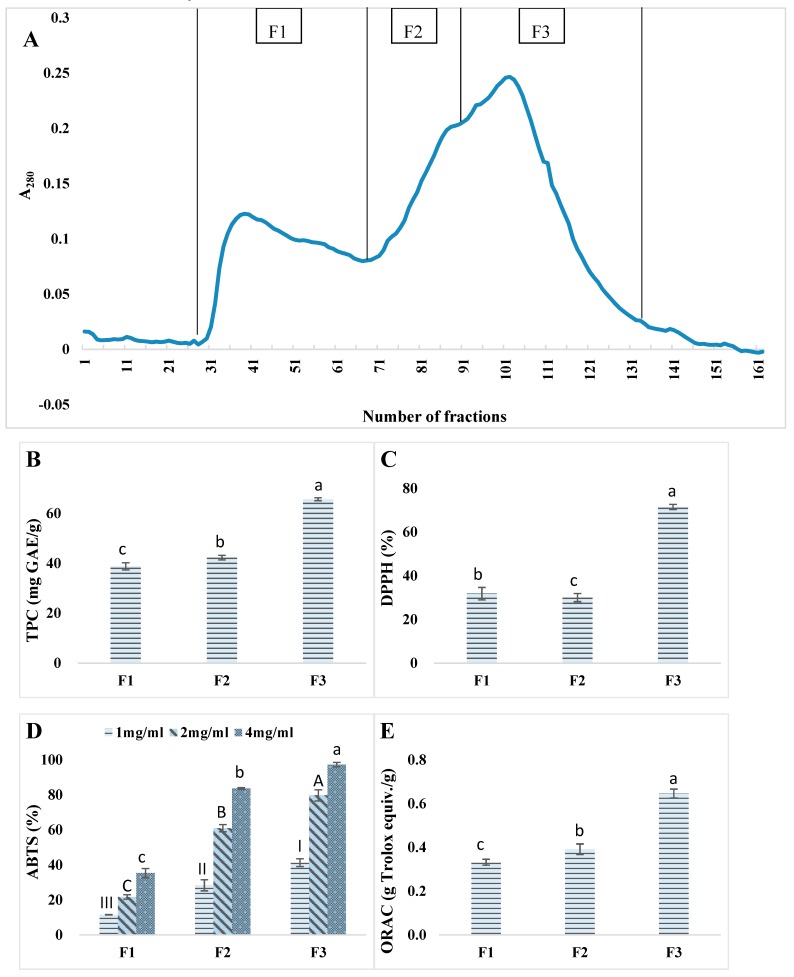
Gel filtration chromatography of kafirin Alcalase 5–10 kDa hydrolysates prepared at enzyme-to-substrate ratio of 0.4 Au/g and hydrolyzed for 4 h on a Sephadex G-25 column (26 mm × 850 mm). (**A**). Gel filtration chromatogram (Abs at 280 nm); (**B**). Total phenolic content (mg GAE/g); (**C**). DPPH scavenging activity (%) at 5 mg/mL; (**D**). ABTS scavenging activity (%); (**E**). ORAC (g Trolox equivalent/g). Different lowercase and capital letters indicated significant difference at *p* < 0.05.

Kafirin Alcalase 5–10 kDa fraction with relatively higher antioxidant activity was further fractionated on a Sephadex-G25 gel filtration column. The elution profile was divided into three fractions (F1–F3) and each fraction was collected and freeze-dried for analysis of total phenolic content and antioxidant activities (Figure 5). Overall, the smaller sized fraction F3 manifested significantly (*p* < 0.05) higher values in TPC, DPPH%, ABTS%, and ORAC than the other two fractions. F3 also possessed an enhanced antioxidant activity as well as TPC than the hydrolysate mixutre without gel filtration. Thus, gel filtration was considered as an effective approach to fractionte and isolate the most promising portions of peptides from the hydrolysate mixture. F3 was collected for further analyses. 

#### 3.4.2. Identification of Representative Peptide Sequences from Gel Filtration

**Figure 6:**
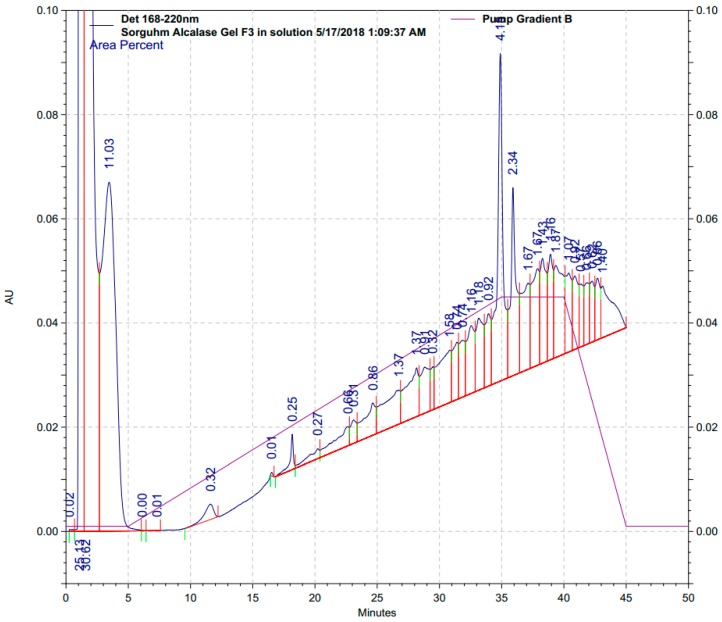
RP-HPLC chromatogram of Fraction 3 from gel filtration of 5–10 kDa kafirin Alcalase hydrolysates prepared at enzyme-to-substrate ratio of 0.4 Au/g and hydrolyzed for 4 h.

The antioxidant activity of protein hydrolysates was dependent upon the characteristic amino acid sequences of the peptides derived [40], as dictated by the protease specificity. To identify the peptide profile present in the fraction of hydrolysates with potent antioxidant activity, F3 from the gel filtration was further isolated and analyzed by RP-HPLC (Figure 6) followed by MALDI-TOF/TOF MS analysis. 

Peaks at 1.6-, 3.6-, 35.0-, and 36.0-min possessing area percentages of 30.62%, 11.03%, 2.34% and 4.15% were collected and analyzed, respectively. A total of 26 peptide sequences were identified, as listed in Table 1. The sequences of peptides are presented in abbreviated amino acid codes in accordance with the International Nucleotide Sequence Database. Alcalase is a serine endopeptidase with broad specificity but a preference for large, uncharged amino acid side-chain groups [11,12]. It was found that, all four peaks from HPLC contained peptide sequences of QQWQ and QWQQ, which could be critical peptide sequences responsible for the antioxidant activity of kafirin Alcalase hydrolysates. Valine (Val, V), leucine (Leu, L) and isoleucine (Ile, I) are hydrophobic amino acids, found to be present in almost all the identified peptide sequences. The presence of these hydrophobic amino acids increased the solubility of their compositional peptides in lipid phase, thus, enhanced their accessibility to the hydrophobic lipid and/or oil targets. Besides, cysteine (Cys, C) and methionine (Met, M) are typical nucleophilic sulfur-containing amino acids that are widely accepted as antioxidant amino acids or important constituent amino acids in antioxidant peptide sequences despite some prooxidant properties under some circumstances [38,41], which were found to be present in 10 and 5 of the identified peptides, respectively. Tryptophan (Trp, W) is an aromatic amino acid that was also reported to be an important antioxidant amino acid due to the hydrogen donating ability of the aromatic ring [42]. 10 of the identified peptides were found to contain Try at terminals or within sequences. 

## 4. Conclusions

Alcalase is an effective enzyme in producing antioxidant peptides from kafirin with a relatively high hydrolysate yield, increased total phenolic content, and enhanced antioxidative activities. Medium-sized fraction of hydrolysates (5–10 kDa) from MWCO membrane filtration possessed the highest total phenolic content, DPPH radical scavenging activity, ABTS radical scavenging activity, reducing power, and metal chelating capacity. The selected fraction of hydrolysates unveiled remarkable inhibition effects against lipid oxidation in both emulsion and ground meat model systems. Smaller-sized fraction (F3) collected through gel filtration chromatography had significantly stronger antioxidant activities than other fractions. By using RP-HPLC followed with MALDI-TOF/TOF MS analysis, 26 representative peptide sequences were identified. Possessing both high antioxidant activity and hydrolysate recovery yield, the novel kafirin hydrolysates obtained with Alcalase are attracting considerations as alternatives to synthetic antioxidants in various food products as multi-functional ingredients (antioxidants, protein nutrients, surface-active agents, etc.).

## Figures and Tables

**Table 1:** Representative antioxidant peptides in kafirin Alcalase hydrolysates.

RP-HPLC Peak	1.6 min	3.6 min	35.0 min	36.0 min
Area%	30.62%	11.03%	2.34%	4.15%
Coverage%	36.50%	82.80%	54.70%	27.10%
Sequences *	KMVIV	QWQQ	QQWQ	KMVIV
	LAVCLA	QQWQ	QWQQ	AVCLAL
	AVCLAL	GVVQSV	GVVQSV	LAVCLA
	QQWQ		QLQGVA	QQWQ
	QWQQ		VQQLQ	QWQQ
	RQQCC		VAQVAQ	
	MCGWQ		RQQCC	
	CATSAAI		MCGWVVQ	
			CATSAAI	
			DMQSR	

Note: * The sequences of peptides are presented in abbreviated amino acid codes in accordance with the International Nucleotide Sequence Database.

## References

[1] Awika J.M. (2011). Major cereal grains production and use around the world. ACS Symp. Ser..

[2] Lee B.H., Weller C.L., Cuppett S.L., Carr T.P., Walter J., Martínez I., Schlegel V.L. (2011). Grain sorghum lipids: Extraction, characterization, and health potential. ACS Symp. Ser..

[3] Brewer M.S. (2011). Natural antioxidants: Sources, compounds, mechanisms of action, and potential applications. Compr. Rev. Food Sci. Food Saf..

[4] Masisi K., Beta T., Moghadasian M.H. (2016). Antioxidant properties of diverse cereal grains: A review on *in vitro* and *in vivo* studies. Food Chem..

[5] Elias R.J., Kellerby S.S., Decker E.A. (2008). Antioxidant activity of proteins and peptides. Crit. Rev. Food Sci. Nutr..

[6] Sarmadi B.H., Ismail A. (2010). Antioxidative peptides from food proteins: A review. Peptides.

[7] Zhao Q., Selomulya C., Wang S., Xiong H., Chen X.D., Li W., Peng H., Xie J., Sun W., Zhou Q. (2012). Enhancing the oxidative stability of food emulsions with rice dreg protein hydrolysate. Food Res. Int..

[8] Jin D.X., Liu X.L., Zheng X.Q., Wang X.J., He J.F. (2016). Preparation of antioxidative corn protein hydrolysates, purification and evaluation of three novel corn antioxidant peptides. Food Chem..

[9] Alashi A.M., Blanchard C.L., Mailer R.J., Agboola S.O., Mawson A.J., He R., Girgih A., Aluko R.E. (2014). Antioxidant properties of Australian canola meal protein hydrolysates. Food Chem..

[10] Xia Y., Bamdad F., Gänzle M., Chen L. (2012). Fractionation and characterization of antioxidant peptides derived from barley glutelin by enzymatic hydrolysis. Food Chem..

[11] Kong B., Xiong Y.L. (2006). Antioxidant activity of zein hydrolysates in a liposome system and the possible mode of action. J. Agric. Food Chem..

[12] Zheng X.Q., Li L.T., Liu X.L., Wang X.J., Lin J., Li D. (2006). Production of hydrolysate with antioxidative activity by enzymatic hydrolysis of extruded corn gluten. Appl. Microbiol. Biotechnol..

[13] Zhu K., Zhou H., Qian H. (2006). Antioxidant and free radical-scavenging activities of wheat germ protein hydrolysates (WGPH) prepared with alcalase. Process Biochem..

[14] Kim S.K., Kim Y.T., Byun H.G., Nam K.S., Joo D.S., Shahidi F. (2001). Isolation and characterization of antioxidative peptides from gelatin hydrolysate of Alaska pollack skin. J. Agric. Food Chem..

[15] Park P.J., Jung W.K., Nam K.S., Shahidi F., Kim S.K. (2001). Purification and characterization of antioxidative peptides from protein hydrolysate of lecithin-free egg yolk. J. Am. Oil Chem. Soc..

[16] Thamnarathip P., Jangchud K., Nitisinprasert S., Vardhanabhuti B. (2016). Identification of peptide molecular weight from rice bran protein hydrolysate with high antioxidant activity. J. Cereal Sci..

[17] Agrawal H., Joshi R., Gupta M. (2017). Isolation and characterisation of enzymatic hydrolysed peptides with antioxidant activities from green tender sorghum. LWT—Food Sci. Technol..

[18] Zhang L., Li J., Zhou K. (2010). Chelating and radical scavenging activities of soy protein hydrolysates prepared from microbial proteases and their effect on meat lipid peroxidation. Bioresour. Technol..

[19] Li Y., Jiang B., Zhang T., Mu W., Liu J. (2008). Antioxidant and free radical-scavenging activities of chickpea protein hydrolysate (CPH). Food Chem..

[20] Kou X., Gao J., Zhang Z., Wang H., Wang X. (2013). Purification and identification of antioxidant peptides from chickpea (*Cicer arietinum* L.) albumin hydrolysates. LWT—Food Sci. Technol..

[21] Pan M., Jiang T.S., Pan J.L. (2011). Antioxidant activities of rapeseed protein hydrolysates. Food Bioprocess Tech..

[22] Bougatef A., Nedjar-Arroume N., Manni L., Ravallec R., Barkia A., Guillochon D., Nasri M. (2010). Purification and identification of novel antioxidant peptides from enzymatic hydrolysates of sardinelle (*Sardinella aurita*) by-products proteins. Food Chem..

[23] Moraes É.A., Marineli R.D.S., Lenquiste S.A., Steel C.J., de Menezes C.B., Queiroz V.A.V., Maróstica Júnior M.R. (2015). Sorghum flour fractions: Correlations among polysaccharides, phenolic compounds, antioxidant activity and glycemic index. Food Chem..

[24] Wu Q., Du J., Jia J., Kuang C. (2016). Production of ACE inhibitory peptides from sweet sorghum grain protein using alcalase: Hydrolysis kinetic, purification and molecular docking study. Food Chem..

[25] Ortíz Cruz R.A., Cárdenas López J.L., González Aguilar G.A., Astiazarán García H., Gorinstein S., Canett Romero R., Robles Sánchez M. (2015). Influence of sorghum kafirin on serum lipid profile and antioxidant activity in hyperlipidemic rats (*in vitro* and *in vivo* studies). Biomed Res. Int..

[26] Sullivan A.C., Pangloli P., Dia V.P. (2018). Kafirin from Sorghum bicolor inhibition of inflammation in THP-1 human macrophages is associated with reduction of intracellular reactive oxygen species. Food Chem. Toxicol..

[27] Kamath V., Niketh S., Chandrashekar A., Rajini P.S. (2007). Chymotryptic hydrolysates of α-kafirin, the storage protein of sorghum (*Sorghum bicolor*) exhibited angiotensin converting enzyme inhibitory activity. Food Chem..

[28] Wang Y., Tilley M., Bean S., Susan Sun X., Wang D. (2009). Comparison of methods for extracting kafirin proteins from sorghum distillers dried grains with solubles. J. Agric. Food Chem..

[29] Nielsen P.M., Petersen D., Dambmann C. (2001). Improved method for determining food protein degree of hydrolysis. J. Food Sci..

[30] Huang D., Ou B., Hampsch-Woodill M., Flanagan J.A., Deemer E.K. (2002). Development and validation of oxygen radical absorbance capacity assay for lipophilic antioxidants using randomly methylated β-cyclodextrin as the solubility enhancer. J. Agric. Food Chem..

[31] Duh P.D., Tu Y.Y., Yen G.C. (1999). Antioxidant activity of water extract of harng jyur (*Chrysanthemum morifolium Ramat*). LWT—Food Sci. Technol..

[32] Decker E.A., Welch B. (1990). Role of ferritin as a lipid oxidation catalyst in muscle food. J. Agric. Food Chem..

[33] Faraji H., McClements D.J., Decker E.A. (2004). Role of continuous phase protein on the oxidative stability of fish oil-in-water emulsions. J. Agric. Food Chem..

[34] Zhang H., Yu L., Yang Q., Sun J., Bi J., Liu S., Zhang C., Tang L. (2012). Optimization of a microwave-coupled enzymatic digestion process to prepare peanut peptides. Molecules.

[35] Liu F., Chen Z., Shao J., Wang C., Zhan C. (2017). Effect of fermentation on the peptide content, phenolics and antioxidant activity of defatted wheat germ. Food Biosci..

[36] Wang X., Zheng X., Kopparapu N., Cong W., Deng Y., Sun X., Liu X. (2014). Purification and evaluation of a novel antioxidant peptide from corn protein hydrolysate. Process Biochem..

[37] Tang N., Zhuang H. (2014). Evaluation of antioxidant activities of zein protein fractions. J. Food Sci..

[38] Wang J., Zhao M., Zhao Q., Jiang Y. (2007). Antioxidant properties of papain hydrolysates of wheat gluten in different oxidation systems. Food Chem..

[39] Cheng Y., Xiong Y.L., Chen J. (2010). Antioxidant and emulsifying properties of potato protein hydrolysate in soybean oil-in-water emulsions. Food Chem..

[40] Chen H.M., Muramoto K., Yamauchi F. (1995). Structural analysis of antioxidative peptides from soybean β-conglycinin. J. Agric. Food Chem..

[41] Samaranayaka A.G.P., Li-Chan E.C.Y. (2011). Food-derived peptidic antioxidants: A review of their production, assessment, and potential applications. J. Funct. Foods.

[42] Guo H., Kouzuma Y., Yonekura M. (2009). Structures and properties of antioxidative peptides derived from royal jelly protein. Food Chem..

